# Use of Large and Diverse Datasets for ^1^H NMR Serum Metabolic Profiling of Early Lactation Dairy Cows

**DOI:** 10.3390/metabo10050180

**Published:** 2020-04-30

**Authors:** Timothy D. W. Luke, Jennie E. Pryce, Aaron C. Elkins, William J. Wales, Simone J. Rochfort

**Affiliations:** 1 Agriculture Victoria Research, AgriBio, Centre for AgriBioscience, Bundoora 3083, Australia; tim.luke@agriculture.vic.gov.au (T.D.W.L.); jennie.pryce@agriculture.vic.gov.au (J.E.P.); aaron.elkins@agriculture.vic.gov.au (A.C.E.); 2 School of Applied Systems Biology, La Trobe University, Bundoora 3083, Australia; 3 Agriculture Victoria Research, Ellinbank Centre, Ellinbank 3821, Australia; bill.wales@agriculture.vic.gov.au

**Keywords:** NMR, metabotype, metabolomics, transition, ketosis, cattle, chemometrics, spectral correction

## Abstract

Most livestock metabolomic studies involve relatively small, homogenous populations of animals. However, livestock farming systems are non-homogenous, and large and more diverse datasets are required to ensure that biomarkers are robust. The aims of this study were therefore to (1) investigate the feasibility of using a large and diverse dataset for untargeted proton nuclear magnetic resonance (^1^H NMR) serum metabolomic profiling, and (2) investigate the impact of fixed effects (farm of origin, parity and stage of lactation) on the serum metabolome of early-lactation dairy cows. First, we used multiple linear regression to correct a large spectral dataset (707 cows from 13 farms) for fixed effects prior to multivariate statistical analysis with principal component analysis (PCA). Results showed that farm of origin accounted for up to 57% of overall spectral variation, and nearly 80% of variation for some individual metabolite concentrations. Parity and week of lactation had much smaller effects on both the spectra as a whole and individual metabolites (<3% and <20%, respectively). In order to assess the effect of fixed effects on prediction accuracy and biomarker discovery, we used orthogonal partial least squares (OPLS) regression to quantify the relationship between NMR spectra and concentrations of the current gold standard serum biomarker of energy balance, β-hydroxybutyrate (BHBA). Models constructed using data from multiple farms provided reasonably robust predictions of serum BHBA concentration (0.05 ≤ RMSE ≤ 0.18). Fixed effects influenced the results biomarker discovery; however, these impacts could be controlled using the proposed method of linear regression spectral correction.

## 1. Introduction

Modern metabolomic techniques such as proton nuclear magnetic resonance (^1^H NMR) spectroscopy allow high-throughput, synchronous characterization of the small metabolites present in biological matrices [1]. In dairy cows, the metabolome gives a snapshot of the complex interactions between host genetics, the rumen microbiome, and the environment at a given time point. ^1^H NMR-based metabolomics therefore offers exciting opportunities to better understand and characterize the complex physiological and biochemical challenges facing cows in the transition period (defined as the three weeks before and after calving [2,3]) which is the period of greatest disease risk [4]. This in turn can facilitate identification of new molecular phenotypes (metabotypes) for genetic selection for improved animal health. These “intermediate phenotypes,” so-termed because they sit between the genome and external phenotype [5], can then be integrated with genomic data to improve genomic prediction accuracies of complex traits [6,7]. The aim of metabotype identification is therefore to identify biomarkers that represent inter-animal variation free of confounding environmental factors.

Another aim of dairy cattle metabolomic studies is to identify biomarkers which enable early identification of health disorders in the transition period such as ketosis [8,9], hypocalcemia [10] and displaced abomasa [11]. Of particular interest are studies that have identified biomarkers that are predictive of transition period disorders, such as that by Hailemariam et al. [12], who identified a panel of three metabolites that could predict the occurrence of peri-parturient disease up to four weeks before calving. If robust, such predictive biomarkers would enable producers and veterinarians to implement preventive nutritional, management and/or veterinary interventions before the onset of disease.Unlike metabotype biomarkers used for genetic selection, the aim of biomarkers used for management purposes is to predict the external phenotype, and these must therefore capture all sources of phenotypic variation (i.e., host genetics, rumen microbiome, and the environment).

To date, most serum ^1^H NMR-based metabolomic studies of livestock have involved relatively small numbers of animals, often of a single breed, and often located on a single farm. In their review, Goldansaz et al. [13] identified limited sample size and diversity as limitations of many livestock metabolomics studies and highlighted the need for larger and more diverse datasets to ensure models and biomarkers are robust. However this needs to be balanced against the need for careful experimental design to account for potential confounding from systematic environmental effects such as diet/nutritional management, parity and stage of lactation, which are known to affect the metabolic status of cows [13]. However, in order to achieve large datasets, it may be necessary to obtain samples from multiple different farms, especially when the prevalence of the condition being investigated is low (e.g., displaced abomasa). Previous studies have reported differences in the milk metabolome of animals from different geographical regions [14], farms [15], and of different breeds [16]. However, given that there is not a strong relationship between blood and milk metabolomes [17,18], these findings cannot be extrapolated to the blood serum/plasma metabolome. More information is therefore needed on the impact of systematic environmental effects on the serum metabolome of livestock.

Linear models are routinely used by quantitative geneticists to account for the influence of systematic environmental effects (also known as fixed effects) known to have significant effects on phenotypic variation [19], and thus disentangle genetic from non-genetic effects. Frequently used fixed effects include stage of lactation, parity, and herd-year-season. Similar approaches have recently been applied to metabolomic data, for example Wanichthanarak et al. [20], who used linear mixed-effects models and patient metadata to account for biological variation in metabolomics data, and Laine et al. [21], who used linear models to study the effect of pregnancy on mid-infrared spectral data derived from cows’ milk. 

The aim of this study was therefore to investigate the feasibility of using of large and diverse datasets in livestock metabolomics studies by examining the effects of fixed environmental and physiological effects on the ^1^H NMR serum metabolome of clinically healthy dairy cows in early lactation. We propose a method that uses linear models to correct spectra for fixed effects and demonstrate its potential utility by quantifying the relationship between ^1^H NMR spectra and the current gold-standard serum biomarker of energy balance, β-hydroxybutyrate (BHBA) [22,23]. 

## 2. Results

### 2.1. Dataset

Serum samples were collected from 707 early lactation cows (<30 d in milk) from 13 farms located in southeastern Australia. Descriptive statistics of the animals included in the experiment, including herd of origin, stage of lactation (reported as days in milk, or the number of days post-calving), parity and serum BHBA concentrations and are summarized in Table 1. Of particular interest were the BHBA results obtained from Farm 1, which had a greater mean and standard deviation than observed in other farms.

### 2.2. ^1^H NMR Spectroscopy of Serum Samples

^1^H NMR spectra were complex; however, more than 20 metabolites could be identified. Spectra were dominated by organic acids, amino acids, glucose and phospholipid intermediates (Figure S1 and Table S1). 

### 2.3. Preliminary Data Analysis Using Principal Component Analysis

Preliminary data analysis and outlier identification was performed using principal component analysis (PCA). Plots of the first 2 principal components (PCs) identified several samples located outside the 95% confidence level. These spectra were manually inspected, and a single outlier with erroneous phasing was identified and removed from subsequent analyses.

**Figure 1:**
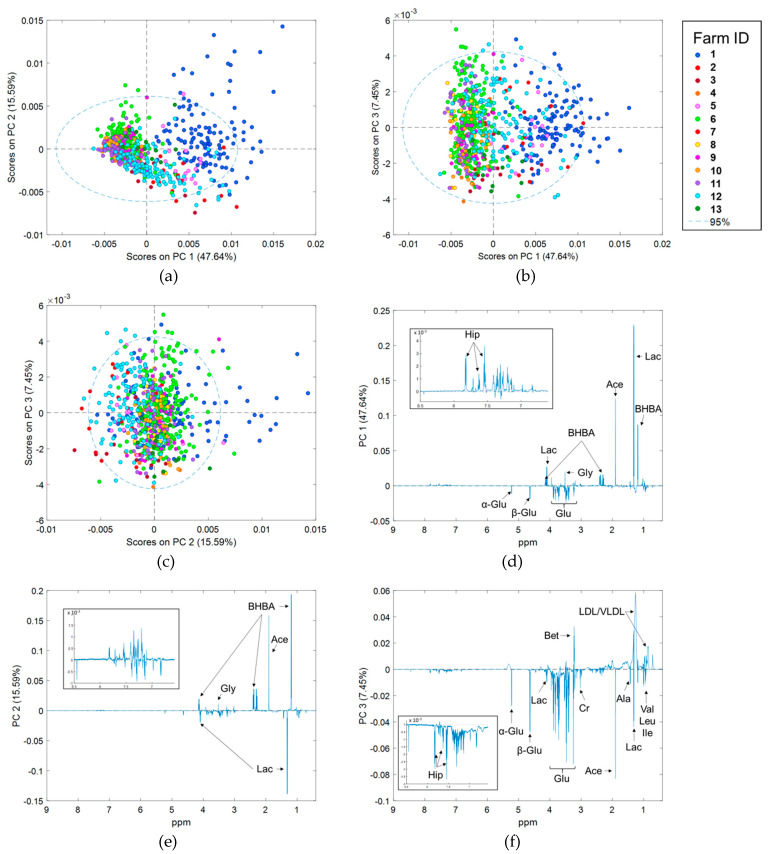
Results of principal component analysis (PCA) of 707 proton nuclear magnetic resonance (^1^H NMR) spectra of serum obtained from dairy cows in early lactation; (**a**) principal component (PC) 1 vs. PC 2 scores, (**b**) PC 1 vs. PC 3 scores, (**c**) PC 2 vs. PC 3 scores, (**d**) PC 1 loadings, (**e**) PC 2 loadings, and (**f**) PC 3 loadings plots. Scores plots are colored by farm of origin. The δ 6.5 to 8.5 region of loadings plots have been magnified for clarity purposes. α-Glu = α glucose, Ace = acetate, Ala = alanine, β-Glu = β glucose, Bet = betaine, BHBA = β hydroxybutyrate, Cr = creatine, Glu = glucose, Gly = glycine, Hip = hippurate, Ile = isoleucine, Lac = lactate, Leu = leucine, Val = valine, VLDL/LDL = Very low density lipoprotein and low density lipoprotein.

PCA was repeated after outlier removal. The first 13 PCs explained greater than 90% of the variation in the spectra. Scores plots of the first three PCs, which explain 47.64%, 15.59%, and 7.45% of variation, respectively, are shown in Figure 1a–c. There was obvious clustering of samples by herd of origin. Samples from Farm 1 showed greater variation than those from the other farms. The separation between farm clusters was most obvious along PC1 and PC2. Visual comparisons based on stage of lactation (defined as weeks in milk (WIM)) and parity were also performed, but no obvious clustering or separation was observed. Loadings plots of the first three PCs show that energy metabolites BHBA, lactate, acetate and glucose, have the largest influences on spectral differences (Figure 1d–f), with smaller influences from the branched chain amino acids, lipoproteins, glycine, creatine, and betaine.

### 2.4. Principal Component Analysis of Spectra Corrected for Fixed Effects

**Figure 2:**
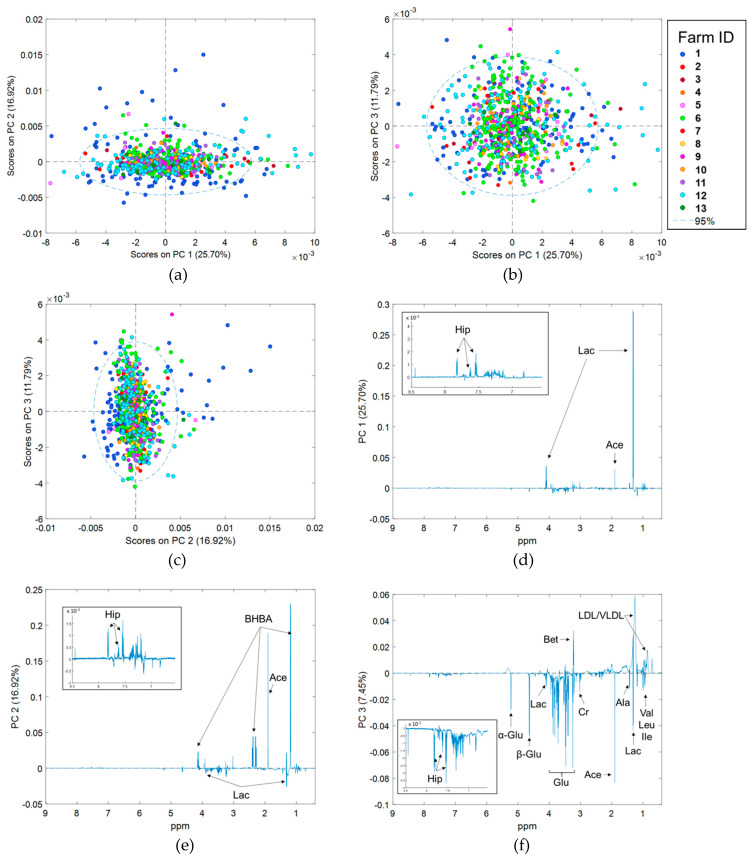
Results of PCA of 707 ^1^H NMR spectra of serum, corrected for herd of origin, week of lactation, and parity obtained from dairy cows in early lactation; (**a**) PC 1 vs. PC 2 scores, (**b**) PC 1 vs. PC 3 scores, (**c**) PC 2 vs. PC 3 scores, (**d**) PC 1 loadings, (**e**) PC 2 loadings, and (**f**) PC 3 loadings plots. Scores plots are colored by farm of origin. The δ 6.5 to 8.5 region of loadings plots have been magnified for clarity purposes. α-Glu = α glucose, Ace = acetate, Ala = alanine, β-Glu = β glucose, Bet = betaine, BHBA = β hydroxybutyrate, Cr = creatine, Glu = glucose, Gly = glycine, Ile = isoleucine, Lac = lactate, Leu = leucine, Val = valine, VLDL/LDL = Very low density lipoprotein and low density lipoprotein.

Principal component analysis (PCA) was repeated on spectra that had been corrected for (1) WIM, (2) Parity, (3) Herd, and (4) WIM, Parity and Herd simultaneously (hereafter referred to as all fixed effects) (Models 1 to 4). Results derived from spectra corrected separately for WIM and Parity are nearly identical to uncorrected spectra (Figures S2 and S3). By contrast, scores plots derived from PCA of spectra corrected for Herd (Figure S4), and spectra corrected for all fixed effects (Figure 2a–c), show no obvious clustering of samples by Herd, WIM or Parity. There is, however, still considerable separation of samples along all three PC axes, suggesting that significant inter-animal variation in the serum metabolome exists after accounting for fixed effects. Compared to the uncorrected data; (1) more PCs were required to explain >90% of spectral variation (24 vs. 13), (2) the percentage of variation captured by PC1 was lower (25.70% vs. 47.64%), and (3) the percentage of variation captured by PC2 and PC3 was higher (16.92% vs. 15.59% and 11.79% vs. 7.45%, respectively). Loadings plots are shown in Figure 2d–f. Interestingly, separation of samples along PC1 (25.70%) is due almost entirely to lactate. Loadings on PC2 and PC3 are similar to uncorrected data.

### 2.5. Effect of Stage of Lactation, Parity, and Herd Effects on ^1^H NMR Spectra 

**Figure 3:**
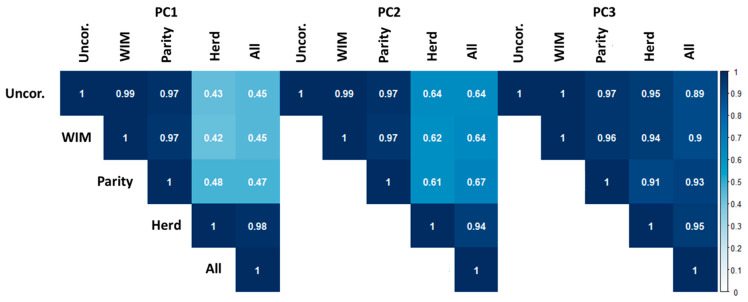
Pearson’s correlations between scores derived from PCA of uncorrected ^1^H NMR spectra of bovine serum, and the same spectra corrected using linear regression for week of lactation (WIM), parity, herd of origin, and WIM, parity, and herd simultaneously (All). Color map shows strength of Pearson’s correlation.

In order to quantify the effect of each fixed effect on NMR spectra, we calculated Pearson’s correlations between scores for the first three PCs from the previously described PCAs (Figure 3). The largest differences (i.e., lowest correlations) were seen between uncorrected spectra, and spectra corrected for Herd (r = 0.43). This suggests that there are significant differences between those 2 spectral datasets, and that Herd, therefore, has a significant effect on the serum NMR metabolome. This is consistent with the clustering of samples by farm in the original PCA (Figure 1a–c). By comparison, the correlations between PC scores derived from uncorrected spectra, and spectra corrected for WIM and Parity, were high (0.99 and 0.97, respectively). This suggests that these spectra are nearly identical, and that these fixed effects have minimal influence on the serum metabolome. Correlations between PC2 scores were consistent with those observed between PC1 scores, and correlations between PC3 scores were all high (≥0.89). 

To test the statistical significance of fixed effects on ^1^H NMR spectra, we used conditional Wald F statistics derived from multiple linear regression models on the first three PC scores (Model 5). The higher the F statistic, the greater the effect of that variable on the PC score, and the lower the P value, the greater the statistical significance. Results derived from these models are summarized in Table 2. PC1 results were consistent with the results of Pearson’s correlations, showing that Herd had the greatest effect. Interestingly, results for PC2 and PC3 differed slightly from Pearson’s correlations. While Herd had a relatively large and significant (P < 0.001) impact on both PC2 and PC3, the effect of Parity was nearly as great on PC2 scores and greater on PC3 scores.

**Figure 4:**
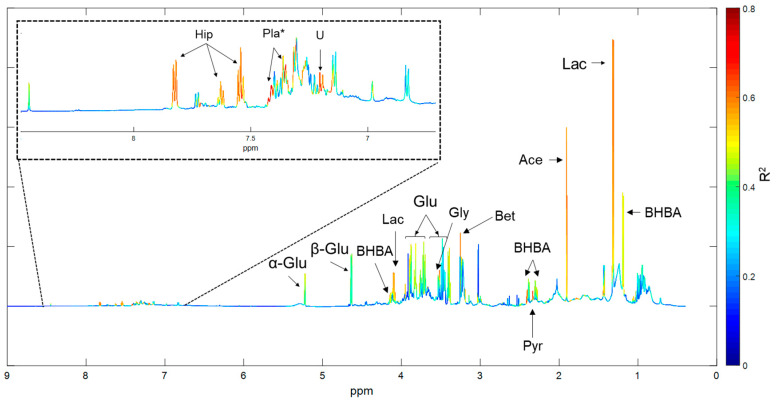
Average ^1^H NMR spectrum of bovine serum. Color coding represents the percentage of variation in the signal at each chemical shift intensity that can be explained by herd of origin. The δ 6.5 to 8.5 region has been magnified for clarity purposes. Ace = acetate, Bet = betaine, BHBA = β hydroxybutyrate, Gly = glycine, Hip = hippurate, Lac = lactate, Pla = 3-phenyllactate, Pyr = pyruvate, U = unidentified peak. * indicates tentative identification.

R^2^ values obtained from models 1–4 were used to investigate which regions of the NMR spectra were most strongly influenced by the fixed effects. As the signal intensity at each chemical shift was treated as a separate response variable, the R^2^ values from Models 1, 2, and 3 describe the effect of WIM, parity, and herd on each of the 24,349 chemical shifts, respectively. These R^2^ values were color-coded, and overlaid on an average NMR spectrum. Plots showing the effects of WIM and Parity were unremarkable (all R^2^ < 0.2, Figure S5), however R^2^ values obtained from Model 2 showed that approximately 10–20% of the variation in glucose and acetate concentration could be explained by parity. The plot showing the effect of herd is shown in Figure 4. The strongest effect was seen in the downfield region of the spectrum, with close to 80% of variation in the concentration of some phenolic compounds being explained by Herd. Of these, hippurate could be clearly identified. Peaks at δ 7.31 and 7.39 were tentatively assigned to 3-phenyllactate, but the peak at δ 7.22 could not be identified. Lactate, acetate, BHBA, betaine, pyruvate, glycine, and glucose concentrations were also strongly influenced by herd effect.

The results of ANOVA-simultaneous component analysis (ASCA) were consistent with results of linear regression spectral correction and are shown in Table S2. Herd had the greatest effect (43.99, P = 0.02), followed by parity (4.10, P = 0.02) and WIM (1.37, P = 0.02). When ASCA was performed on corrected spectra, the effect of the fixed effect(s) was reduced to zero. For example, when ASCA was performed on spectra corrected for Herd, the effect of herd was zero (P = 1.00), but the effects of WIM (1.68, P = 0.02) and parity (3.30, P = 0.02) were retained.

### 2.6. Robustness of ^1^H NMR Predictions of Serum BHBA

Our results show that ^1^H NMR spectra can be used to predict serum BHBA concentration with good accuracy. This result is expected, as BHBA is directly quantifiable from NMR spectra. The overall robustness of our approach was assessed using a “leave-one-farm-out” external validation of OPLS models built using uncorrected data. This involved iteratively setting aside data from one farm, training models using data from the remaining 12 farms, then using the withheld data for external validation. R^2^ results were variable (0.30 ≤ R^2^ ≤ 0.99), however RMSE values remained relatively low (≤ 0.18) (Table 3). Interestingly, RMSE values were considerably higher when Farm 1 data were withheld for validation.

### 2.7. Influence of Fixed Effects on Interpretation of ^1^H NMR Metabolomic Data

The impact of fixed effects on the interpretation of ^1^H NMR metabolomic data was determined by comparing the results of OPLS models built using (1) data from Farm 1 only (used as a control), (2) uncorrected data from all farms, and (3) data from all farms corrected for all fixed effects. Fixed effects appeared to have minimal effect on the predictive ability of models. We observed similar 10-fold cross validation prediction accuracies for all 3 datasets (Table 4). Interestingly, RMSE results were quite close to the results of the leave-one-farm out external validation (0.05 ≤ RMSE ≤ 0.18).

**Figure 5:**
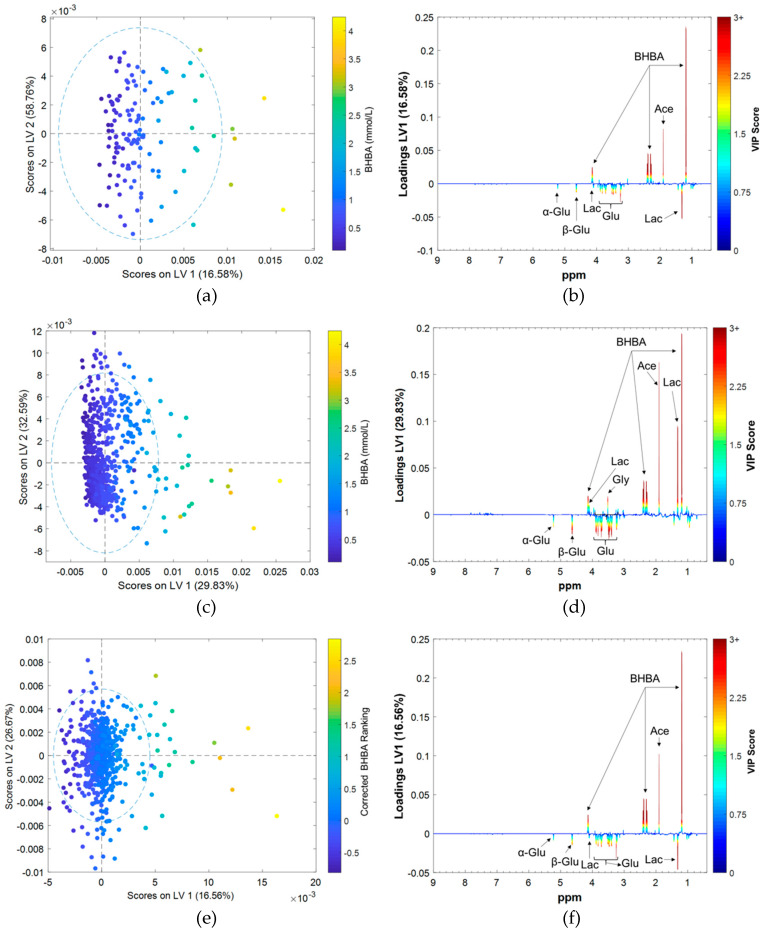
Results of OPLS regressions of serum BHBA concentration against ^1^H NMR spectrum of bovine serum: (**a**) Farm 1 (N = 179) LV1 vs. LV2 scores and (**b**) LV1 loadings, (**c**) all farms (N = 707) uncorrected data LV1 vs. LV2 scores and (**d**) LV1 loadings, and (**e**) all farms data LV1 vs. LV2 scores and (**d**) LV1 loadings.

The influences of fixed effects on biomarker discovery were investigated by comparing loadings on LV1. Results obtained using only Farm 1 data were used as a reference and show a strong positive correlation between BHBA concentration and acetate, and strong negative correlations with lactate and glucose (Figure 5a,b). Loadings from the complete dataset corrected for all fixed effects were very similar (Figure 5e,f). Results from uncorrected data, however, were quite different (Figure 5c,d), with BHBA being positively correlated with lactate and glycine. Examination of scores plots shows obvious clustering and separation by herd (especially Farm 1) when uncorrected data are used (Figure S6a), but not when corrected data are used (Figure S6b). Results from the original PCA showed that samples from Farm 1 clustered at the positive end of PC1, and that lactate and glycine both had strong positive influences on PC1 loadings. Therefore, it is possible that OPLS results are confounded by a strong herd effect when uncorrected data are used.

## 3. Discussion 

To the best of the authors’ knowledge, this is the first large-scale serum metabolomics study to investigate the impact of systematic environmental and physiological fixed effects on the ^1^H NMR serum metabolome of clinically healthy dairy cattle. Our results indicate that herd-specific environmental factors have much greater effects on the serum metabolome of early lactation dairy cows than physiological factors such as WIM and parity. We demonstrate that, while confounding from herd effects can significantly influence the results of biomarker discovery, models built using data collected from multiple farms can give robust predictions of external phenotypes such as BHBA. In order to overcome the potential confounding of fixed effects on biomarker discovery, we propose a method to correct ^1^H NMR spectra prior to multivariate analysis using multiple linear regression. 

### 3.1. Differences in ^1^H NMR Spectra Between Herds

Our results clearly demonstrate that there are significant differences in the serum metabolomes of animals from different herds. The fact that energy metabolites BHBA, lactate, acetate and glucose dominated PCA loadings (Figure 2d–f), and that herd effect accounted for a large percentage of the variation seen in lactate, acetate, pyruvate, glucose, and BHBA concentrations, (Figure 4) suggests that metabolic state, in particular energy balance, varied significantly between farms. 

The importance of lactate was particularly interesting. Lactate was one of the most abundant metabolites identified in this experiment. This is very different to the findings of Sun et al. [8], who reported that lactate was one of the weakest signals in serum ^1^H NMR spectra obtained from early-lactation cows fed a total mixed ration. One possible explanation for the very high concentrations of lactate seen in our dataset could be ruminal lactate production. During spring, dairy cows in pastoral farming systems of southeastern Australia are typically fed rations high in fermentable carbohydrate, and low in neutral detergent fiber. As a consequence, ruminal acidosis is common [24]. Serum concentrations of lactate, and in particular D-lactate from microbial fermentation, have been shown to increase following experimental induction of ruminal acidosis [25,26]. Without the use of a shift reagent and specialized experiments it is not possible to differentiate between the different lactate isomers by ^1^H NMR [27]. We therefore plan to quantify the relative contributions of L- and D- lactate to better understand the cause of high lactate concentrations in our dataset. 

The strong influence of Herd on the concentration of phenolic compounds could also be consistent with ruminal acidosis. Signal intensities in the downfield region of 2D spectra were weak, meaning clear identification of some of the phenolic peaks in our dataset was not possible. Our tentative identification of 3-phenyllactate is consistent with the findings of Yang et al. [26], who demonstrated that beef steers fed high starch (corn) diets had higher plasma concentrations of phenyllactate compared to those fed low starch diets. This study also identified L-phenyllalanine biosynthesis and metabolism as important metabolic pathways in high starch feeding. We plan to (1) enrich samples and repeat 2D analyses and (2) perform LCMS-based metabolomics on a subset of samples to identify these compounds.

Nearly 80% of the variation seen in hippurate concentration could be explained by herd effect (Figure 4). Hippurate is formed by the conjugation of glycine and benzoic acid, and has been associated with microbial degradation of dietary compounds [28]. Concentrations of hippurate increase with increased consumption of phenolic compounds [13], which are present in relatively high concentrations in pasture species. Milk hippurate concentration has been proposed as a biomarker of pasture/forage intake in goats [29], and it is possible that our results represent differences in feeding regimens between farms. Hippurate has also been proposed as a biomarker for gut microbiome diversity in humans [30], and our results may indicate differences in the gastrointestinal health of animals from different farms (i.e., ruminal acidosis). Detailed information of ration formulations is very difficult to define in grazing systems as pasture quality and intake vary considerably within and between herds. This information was therefore not available for the herds in our dataset and more data are required to further investigate this finding.

Results of the initial PCA showed that data from Farm 1 were significantly different to, and showed more variation than, data from the other farms. The reasons for these differences are hard to determine from our dataset, as Farm 1 differed in environment/management, breed and reference BHBA concentrations (and therefore it is assumed animal metabolic status). Given that we also observed clustering and separation of the 12 Holstein-Friesian herds in the initial PCA (Figure 2), it appears that herd-specific environmental factors have a larger effect on the serum metabolome than breed. However, Liao et al. [31] recently reported clear differences in the serum metabolomes (GC-MS) of three different breeds of beef steers, all the same age, fed the same ration, and managed under the same conditions. Further data are therefore required to investigate if there are differences between the serum metabolomes of different dairy breeds.

Pre-analytical sample handling and processing have been shown to have significant effects on human metabolomic data [32], and considerable efforts are made to streamline and standardize sample collection and processing protocols [33,34,35]. Standardizing protocols in livestock studies provides its own challenges, when relatively large number of samples are being taken at once, often in diverse, challenging and remote locations. While all attempts were made to ensure consistency, there were some unavoidable differences in the way samples from different herds were handled (for example time, between blood sample collection and centrifugation varied from approximately 2–4 h). It is therefore possible that some of the variation between farms seen in our data could be due to pre-analytical sample handling. However, overall our results suggest that metabolomic differences between animals from different farms are due largely to differences in diet/nutritional management. We plan to collect more samples from animals receiving different diets to investigate this further. 

### 3.2. Effect of Lactation Stage and Parity on Serum Metabolome

Our results suggest that stage of lactation appeared to have a minimal effect on the NMR spectra. This is consistent with the findings of Ilves et al. [17] who found that the mass spectrometry (MS) based plasma metabolome of dairy cows was more heavily influenced by animal individuality than by lactation stage. By contrast, several authors report that both the NMR and MS-derived milk metabolome changes across lactation [17,36]. This suggests that blood-based metabolomics may be more suitable for identification of individual animal-specific differences within a population, and therefore provide more robust metabotypes for genetic selection.

Parity appeared to have a small but significant (P < 0.05) effect on the overall ^1^H NMR serum metabolome. We could find no other reports in the literature describing the effect of parity on the entire serum metabolome. However, our results are consistent with other studies that showed parity has a significant effect on the concentration of several metabolites in serum including glucose, creatinine, urea and BHBA [37,38,39]. This suggests that parity should be taken into consideration when undertaking metabolomic studies in dairy cows.

### 3.3. Accuracy of OPLS Models for Predicting Serum BHBA Concentration

Despite the significant influence of fixed effects on the serum metabolome, results obtained from the leave-one-farm out external validation suggest that prediction models constructed with data from multiple farms are quite robust. R^2^ values varied significantly depending on which farm was used for validation (0.30 ≤ R^2^ ≤ 0.99); however, the R^2^ is known to be affected by the range of the dataset, and RMSE is often considered to be a better predictor of model performance [40]. Promisingly, external validation RMSE results (0.05 ≤ R^2^ ≤ 0.18) were close to those obtained from 10-fold cross validation of models built using only Farm 1 data (RMSE = 0.12) and all data (RMSE = 0.10). The fact that prediction errors were highest when Farm 1 data were withheld for validation suggests that the increased variation observed in Farm 1 data represents valuable biological variation rather than confounding/noise. 

Correcting data for fixed effects had very little impact on the predictive ability of OPLS models. Furthermore, when corrected spectra were used, y-values also had to be corrected, making interpretation of phenotypic values difficult. Interestingly, Wanichthanarak, et al. [20] found that “readjusting” mass spectroscopy metabolite signals using patient metadata and linear mixed models improved the sensitivity and specificity of classification of human tissue samples with and without colorectal cancer. Conversely, Posma et al. [41] found that adjusting NMR data for confounding factors lead to a loss of predictive power for cardiovascular risk in a large-scale human NMR metabolomic dataset. Whether using NMR spectra corrected using linear regression will improve the performance of classification models (as opposed to regression against a continuous variable as used in this study) requires further investigation. Overall, our results suggest that models constructed using uncorrected data collated from multiple farms may be appropriate for prediction of external phenotypes which are influenced by both genetic and environmental factors. 

### 3.4. Impact of Fixed Effects on the Interpretation of Metabolomic Data for Biomarker and Metabotype Discovery

Loadings from OPLS models built using uncorrected spectra from Farm 1, and spectra from all farms corrected for fixed effects, were consistent with the literature. BHBA and glucose concentrations have been shown to be negatively correlated in the serum of cows in early lactation dairy cows [42]. L-lactate is an important gluconeogenic substrate in dairy cows [43,44], so it follows that lactate concentration is also negatively correlated with BHBA concentration. Our results are also consistent with the findings of Sun et al. [8] who showed that cows with subclinical (1.2 < BHBA < 2.9 mmol/L) and clinical ketosis (BHBA > 2.9 mmol/L) had lower lactate and glucose concentrations and higher BHBA and acetate concentrations than the healthy controls. 

The fact that loadings were different when uncorrected spectra from all farms were used demonstrates that herd-specific environmental effects can influence the results of biomarker discovery. How significant this is ultimately depends on the research question being asked. If the study aim is to identify biomarkers of external phenotypes (i.e., biomarkers that represent both genetic and environmental factors which are used for management purposes such as disease prediction), then the impact of environmental effects is important and must be captured. However, if the aim is to identify biomarkers indicative of inter-animal differences free of environmental confounding, or to understand biological processes, our results suggest that the influence of environmental effects could lead to erroneous results. This is consistent with the findings of Posma et al. [41] who showed that differences in fixed effects between subjects from the north and south of China explained some metabolite associations, which had previously been attributed to cardiovascular disease risk. This study also reported that adjusting metabolomic data for confounding using an algorithm called Covariate-Adjusted Projection to Latent Structures (CA-PLS) improved model interpretability and led to the identification of more robust biomarkers. Our results are also consistent with other studies that have explored the impacts of data pretreatments on the interpretation of metabolomics data. For example, van den Berg et al. [45] showed that pretreatment methods such as scaling, centering and transformations can greatly affect the outcome of metabolomic analyses (including the biological ranking of important metabolites) and have the potential to enhance biological interpretability. Similarly, Emwas et al. [46] concluded that the choice of spectral processing and post-processing depended on many factors including the aim of the experiment and the quality of data. 

We believe that our approach has particular application in animal breeding, where the aim is to understand the biological processes that underpin economically important traits [47] and to identify metabotypes that represent inter-animal variation independent of confounding from systematic environment effects. Even with the advent of genomic selection, livestock genetic studies require relatively large numbers of animals to ensure there is adequate genetic variation in the study population [48,49]. The same is likely to be true for metabotype discovery studies. Such large datasets can be hard to compile, especially when the trait of interest is difficult and/or expensive to measure. As well as collecting data from multiple farms, another potential solution is data sharing through international collaboration. This is routinely done by geneticists; for example, de Haas et al. [50] used data from Holstein cattle in Europe, North America and Australasia to improve genomic prediction accuracies for feed intake. The ability to correct metabolomic data for factors such as experimental batch, diet, herd, year and season should allow similar collaborations in metabotype studies.

## 4. Materials and Methods 

All procedures undertaken in this study were conducted in accordance with the Australian Code of Practice for the Care and Use of Animals for Scientific Purposes (National Health and Medical Research Council, 2013). Approval to proceed was granted by the Agricultural Research and Extension Animal Ethics Committee of the Department of Jobs, Precincts and Resources Animal Ethics Committee (DJPR, 475 Mickleham Road, Attwood, Victoria 3049, Australia), and the Tasmanian Department of Primary Industries, Parks, Water and Environment (DPIPWE Animal Biosecurity and Welfare Branch, 13 St Johns Avenue, New Town, Tasmania 7008, Australia). AEC project approval codes 2017-05 and 2018-07.

### 4.1. Sample Collection

A single 10 mL blood sample was taken from 708 clinical healthy cows, located on 13 farms in south-eastern Australia between September 2017 and July 2019. All cows had been calved 30 days or less at the time of sampling. Cows on all farms except Farm 1 were Australian Holstein-Friesians, while cows on Farm 1 were crossbred animals (including Holstein–Friesian, Jersey, and Australian Red breeds). All farms operated a feeding system reliant on grazed pasture plus other forages, and concentrates fed in the bail at milking time. 

Blood samples were collected from the coccygeal vein into 10 mL serum clot activator vacutainer tubes (Becton Dickinson, Franklin Lakes, NJ, USA). Samples were allowed to clot at room temperature, before being centrifuged at 1000 g for 20 min at 20 °C. Sera were divided into two aliquots. The first aliquot was refrigerated at 4 °C then shipped on ice to a commercial laboratory for BHBA analysis. The second aliquot was stored at –20 °C until processing for NMR spectroscopy.

### 4.2. Reference BHBA Measurements

Serum BHBA concentrations were determined using a colorimetric enzymatic kinetic assay [51]. All assays were performed by Regional Laboratory Services (Benalla, Victoria, Australia) using a Kone 20 XT clinical chemistry analyzer (Thermo Fisher Scientific, Waltham, MA, USA). The uncertainty of measurement (at a 95% confidence level) was ± 0.060 mmol/L at 0.85 mmol/L.

### 4.3. Chemicals

Methanol (>99.9% pure) and dipotassium hydrogen phosphate (anhydrous) were purchased from Fisher Chemical (Fair Lawn, NJ, USA). Sodium 2,2-dimethyl- 2-silapentane-5-sulfonate (DSS-d6, 98%) and deuterium oxide (D_2_O, 98%) were purchased from Cambridge Isotope Laboratories, Inc. (Tewksbury, MA, USA).

### 4.4. Sample Preparation for NMR Spectroscopy

Serum samples were thawed at room temperature for one hour and were prepared for NMR spectroscopy using a methanol protein precipitation method described by Nagana Gowda and Raftery [52]. Briefly, 300 µL of serum was mixed with 600 µL of methanol, vortexed (Ratek multi tube vortex mixer, MTV1), incubated at –20 °C for 20 min, then centrifuged to pellet proteins (11,360 g, 21 °C, 30 min). A 600 µL aliquot of supernatant was then transferred to a clean 2 mL microcentrifuge tube and dried under vacuum at 21 °C overnight using a SpeedVac Savant SPD 2010 Concentrator (Thermo Fisher Scientific, Waltham, MA, USA). Dried extracts were then reconstituted in a D_2_O phosphate buffer solution (100 mM K_2_HPO_4_) containing 0.25 mM DSS-d6 as an internal standard. A 550 µL aliquot was transferred to 5 mm NMR tube for analysis.

### 4.5. ^1^H NMR Data Acquisition

Routine 1D proton spectra were obtained on a Bruker Ascend 700 MHz spectrometer equipped with cryoprobe and SampleJet automatic sample changer (Bruker Biospin, Rheinstetten, Germany). A Bruker noesypr1d pulse sequence was used over –0.76 ppm to 10.32 ppm spectral range with 256 scans collected after eight dummy scans at 298K, with a total acquisition time of 2.11 seconds per increment and a relaxation delay (D1) of 2.00 seconds. The overall number of data points was 32,768. A line broadening of 0.3 Hz was applied to all spectra prior to Fourier transformation. Spectra were manually phased then baseline corrected in Topspin v.3.6.1 (Bruker Biospin, Rheinstetten, Germany). Samples were referenced to the internal standard (DSS-d6) at δ 0.00.

### 4.6. ^1^H NMR Spectral Processing & Multivariate Statistical Analysis

NMR spectra were imported into MatLab v.R2017b (Mathworks, Natick, WA, USA) using the ProMetab v.1.1 script [53]. Each raw spectrum consisted of 31,313 data points between −0.60 and 10.00 ppm. 

Statistical analyses were performed in MatLab utilizing the PLS Toolbox v. 8.5.2 (Eigenvector Research Inc., Manson, WA, USA). The spectral region containing the residual water peak (δ 4.68–5.00) was removed. Spectra were aligned using the correlation optimized warping algorithm [54] to account for chemical shift drift, then normalized to total signal area to account for inherent concentration differences between samples. After normalization, spectral regions containing methanol (δ 3.32–3.36) and DSS-d6 (δ 0.4–−0.60) peaks, and the non-informative region beyond 9.00 ppm were removed. Finally, spectra were baseline corrected using automatic weighted least squares, and scaled by mean centering. After editing, a total of 24,349 chemical shift datapoints were included in subsequent statistical analyses.

For multivariate analyses, unsupervised principal component analysis (PCA) was used. Peaks of interest were identified using the Chenomx NMR suite software v.8.4 (Chenomx Inc., Edmonton, AB, Canada), comparison to the literature, and 2D NMR analysis.

### 4.7. Correction of ^1^H NMR Spectra for the Effects of Systematic Environemtal and Physiological Effects

In order to investigate the effects on spectra of systematic environmental effects (also known as fixed effects) spectra were “corrected” using linear regression models. When correcting for a single categorical fixed effect, this is equivalent to scaling data using the “class centering” pre-processing step. Rather than mean centering, which involves subtracting the global mean from each variable, class centering subtracts the mean of each class. This allows investigation of intra-class variation by removing the effects of inter-class variation [55]. The advantage of using linear models rather than class centering is that the effect of multiple fixed effects or classes can be modelled simultaneously.

The approach we took was based on the principals of quantitative genetic models, where

Phenotypic observation = environmental effects + genetic effects + residual effects(1)

In this study, we only want to remove the effect of environmental factors (as it is the variation in NMR spectra under genetic influence that we are interested in), so the equation can be further simplified to

Phenotypic observation = genetic effects + residual effects(2)

The “corrected phenotype” (i.e., the phenotypic observation with the effects of the environmental effects removed) is defined as the residuals from the above model. For the purposes of this study each chemical shift was treated as a separate phenotype, with the signal intensity at each chemical shift being an individual phenotypic observation. The “corrected spectra” was a matrix of the residuals of each model. 

A 707 × 24,349 matrix of signal intensities of pre-processed spectra was imported into the R statistical software package v 3.6.2 [56]. Each row in the matrix represented a single sample, and each column represented 1 of the 24,349 chemical shifts between δ 0.40 and δ 8.99 that made up an individual spectrum. The following 4 linear models were applied to each of the 24,349 columns in the matrix (i.e., the signal intensity at each chemical shift was treated as the response variable in a separate regression model):

y_il_ = µ + WIM_i_ + e_il_ (Model 1) (3)

y_jl_ = µ + P_j_ + ej_jl_ (Model 2) (4)

y_kl_ = µ + H_k_ + e_kl_ (Model 3) (5)

y_ijkl_ = µ + WIM_i_ + P_j_ + H_k_ + e_ijkl_ (Model 4) (6)

where y is the signal intensity at a given chemical shift, µ is the mean, WIM is weeks in milk (4 levels, defined as 1, 2, 3, or 4), P is parity (4 levels, defined as 1, 2, 3, or ≥ 4), H is the effect of herd (13 levels, with a range of 9 to 248 cows per herd), and e is the random error term. This resulted in four separate 707 × 24,349 matrices containing spectra corrected for the effects of WIM, parity, herd, and all fixed effects, respectively.

The R^2^ values from each regression model were stored in a separate vector. This resulted in four vectors each containing 24,349 R^2^ values; each value representing the percentage of variation in signal intensity explained by the fixed effect(s) at a given chemical shift.

### 4.8. Quantifying the Effect of Stage of Lactation, Parity and Herd on ^1^H NMR Spectra

A separate PCA was performed on each of the 4 corrected spectral datasets (as described in 4.7). Scores of the first three PCs were extracted for each model, and for the PCA model constructed using uncorrected data. We then calculated Pearson’s correlations between scores derived from the 5 PCAs using the corrplot package [57] in R v 3.6.2 [56]. This resulted in three correlation matrices (one for each PC). The lower the Pearson’s correlation coefficient, the greater the differences between PC scores, the greater the differences between the two spectral datasets and therefore the greater the significance of the fixed effect(s).

An alternative approach to investigating the influence of fixed effects is to use multiple linear regression on PC scores from uncorrected spectra. The advantage of this approach is that all fixed effects can be fitted simultaneously, and the statistical significance of each fixed effect can be calculated. The model used was

y_ijkl_ = µ + WIM_i_ + P_j_ + H_k_ + e_ijkl_ (Model 5) (7)

where y is the PC score (on either PC1, PC2, or PC3) and µ, WIM, P, H, and e are the mean, fixed effect, and error terms described previously. The statistical significance of each fixed effect was determined using conditional Wald F statistics in ASReml v 4.2 (VSN International Ltd., Hemel Hempstead, UK). Conditional F statistics are used in multiple linear regression to infer the significance of a given fixed effect assuming that the effect of remaining predictor variables have been accounted for [58].

Finally, we validated our results using the analysis of variance (ANOVA) simultaneous component analysis (ASCA) method in the PLS Toolbox [55]. ASCA is a generalization of ANOVA used to quantify the variation induced by fixed experimental design factors on complex multivariate datasets [59]. ASCA was performed on all spectral datasets (corrected and uncorrected). Statistical significance was determined using permutation testing (50 iterations). 

### 4.9. The Relationships between ^1^H NMR Spectra and Existing Energy Balance Biomarker Concentrations

In order to assess the utility of large and diverse datasets in livestock metabolomics studies, we used orthogonal partial least squares (OPLS) regression to compare ^1^H NMR spectra to serum BHBA concentrations determined by colorimetric assay. The aims of this analysis were (1) to assess the robustness of OPLS models built using uncorrected data and (2) investigate the influence of systematic environmental effects on the interpretation of ^1^H NMR spectra when used for untargeted metabolomic analyses.

#### 4.9.1. Robustness of OPLS Models to Predict External Phenotypes Using Uncorrected Data

The robustness of OPLS models constructed using large and diverse datasets was assessed using a leave-one-farm-out external validation. This involved setting aside data from one farm, training OPLS models using data from the remaining 12 farms, then using the withheld data for external validation. This process was repeated until data from each farm was used as an external validation set once. Model performance was assessed using the R^2^ and RMSE of calibration, cross validation (venetian blind CV with 10 data splits, and one sample per split), and external validation. The statistical significance of OPLS models was determined using permutation testing (cross validated, Wilcoxon test). Only uncorrected data were used for this part of the analysis.

#### 4.9.2. Influence of Fixed Effects on Interpretation of ^1^H NMR Metabolomic Data

To assess the impact of fixed effects on the results of untargeted metabolomic analyses we compared the results of OPLS models constructed from (1) uncorrected data from Farm 1 only (N = 129), (2) uncorrected data from all farms, and (3) data from all farms corrected for all fixed effects (Model 4). Farm 1 data was used to simulate a more “typical” metabolomics experiment in which confounding from environmental effects is controlled through experimental design. 

When corrected spectra were used, reference BHBA concentrations were corrected for the same fixed effects (Model 4). The residuals of this model represent the “corrected BHBA” concentration, which is the expected BHBA concentration of an individual accounting for differences in WIM, Parity and Herd. This poses some challenges in terms of interpretation, as negative residual values (i.e., negative BHBA concentrations) are possible. However, for the purposes of genetic evaluations, the ranking of an animal, or the relative phenotypic value, is of more interest than an absolute value. The corrected value can therefore be considered a “corrected phenotypic ranking.”

The impact of fixed effects on the ability of NMR spectra to predict external phenotypes (i.e., to classify animals or predict biomarker concentrations for management purposes) was assessed by comparing the predictive ability of OPLS models. The influences of fixed effects on biomarker discovery were investigated using scores and loadings on LV1 which show the magnitude and direction of relationships between BHBA concentration and ^1^H NMR spectral features. Variable importance of projection (VIP) scores were used to identify the most statistically significant spectral features in each model. Variables with VIP scores greater than one were considered significant [60].

## 5. Conclusions

In this study we investigated the feasibility of using large and diverse datasets for untargeted ^1^H NMR serum metabolomic profiling of clinically healthy dairy cows in early lactation. In particular, we investigated the effects of systematic environmental factors on the serum metabolome. We used linear regression to correct spectra for (1) herd of origin; (2) parity; (3) WIM; and (4) herd, parity, and WIM simultaneously. Corrected and uncorrected spectra were then analyzed using PCA. Comparison of PCA results showed that herd of origin had a much greater impact on the serum metabolome than either parity or WIM. In order to simulate the impact of these effects in untargeted metabolomics, we used OPLS regression to quantify the relationship between both corrected and uncorrected NMR spectra, and the current gold-standard biomarker of energy balance in dairy cows, BHBA. Our results showed that (1) models constructed using uncorrected data from multiple farms provided reasonably robust predictions of serum BHBA concentration, (2) environmental effects can alter the results of biomarker discovery, and (3) that correcting spectra for environmental effects using linear regression may be useful when the aim of analysis is to investigate phenotypic variation free of confounding from environmental effects (e.g., identification of metabotypes for genetic selection).

## Figures and Tables

**Table 1:** Descriptive statistics of dataset used in this experiment, including farm details, number of cows (N), mean and standard deviation (shown in parentheses) of parity, days in milk (DIM), and serum β hydroxybutyrate (BHBA) concentrations obtained from dairy cows in the first 30 days of lactation from 13 farms in south eastern Australia.

Farm	N	Location	Parity	DIM	BHBA
1	129	Sth Gipp ^1^	2.9 (1.1)	19.4 (7.2)	1.25 (0.69)
2	11	Sth Gipp	2.6 (1.2)	20.4 (8.1)	0.34 (0.12)
3	12	W Gipp ^2^	2.6 (1.4)	22.8 (5.7)	0.33 (0.10)
4	11	W Gipp	3.1 (1.2)	17.9 (10.2)	0.54 (0.15)
5	18	MID ^3^	2.9 (1.1)	22.6 (5.1)	0.61 (0.25)
6	248	W Gipp	2.1 (1.0)	16.7 (6.0)	0.55 (0.21)
7	9	GV ^4^	2.6 (1.0)	13.9 (6.7)	0.53 (0.27)
8	24	MID	2.4 (1.2)	17.7 (8.2)	0.38 (0.09)
9	33	Sth Gipp	2.5 (1.2)	18.3 (7.2)	0.55 (0.33)
10	27	Sth Gipp	1.8 (1.1)	13.1 (7.7)	0.50 (0.14)
11	50	Tas ^5^	2.6 (1.3)	18.6 (7.3)	0.42 (0.17)
12	123	MID	2.8 (1.2)	15.8 (8.6)	0.38 (0.15)
13	12	Tas	2.7 (0.8)	16.0 (7.6)	0.58 (0.22)
ALL	707	-	2.5 (1.2)	17.4 (7.3)	0.63 (0.46)

^1^ South Gippsland Region, ^2^ West Gippsland Region, ^3^ Macalister Irrigation District, ^4^ Goulburn Valley Region, ^5^ Tasmania.

**Table 2:** Results of multiple linear regression models of principal component (PC) scores derived from PCA of ^1^H NMR spectra, against weeks in milk (WIM), parity, and herd of origin. Conditional Wald F statistics (F-con) and corresponding *P* values describe the magnitude and statistical significance of each fixed effect, respectively.

	PC1 (47.64%)			PC2 (15.59%)			PC3 (7.45%)	
Fixed Effect	F-con	*P* Value		F-con	*P* Value		F-con	*P* Value
WIM	2.66	0.047		5.42	0.001		2.14	0.094
Parity	2.78	0.041		20.39	<0.001		15.19	<0.001
Herd	158.29	<0.001		26.78	<0.001		6.66	<0.001

**Table 3:** Results of leave-one-farm out external validation of orthogonal partial least squares (OPLS) regression models predicting serum BHBA concentration from uncorrected ^1^H NMR spectra. Validation farm specifies the identity of the data used for validation, N the number of animals used in calibration and validation datasets. The coefficient of determination (R^2^) and root mean square error (RMSE) are reported for each calibration/validation subset.

			Calibration				Cross Validation			External Validation		
Validation Farm	P	LV	N	R^2^	RMSE		R^2^	RMSE		N	R^2^	RMSE
-	<0.05	3	707	0.95	0.10		0.95	0.10		-	-	-
1	<0.05	5	578	0.87	0.08		0.85	0.08		129	0.96	0.18
2	<0.05	3	696	0.95	0.10		0.95	0.10		11	0.59	0.10
3	<0.05	4	695	0.96	0.09		0.96	0.10		12	0.78	0.06
4	<0.05	3	696	0.95	0.10		0.95	0.10		11	0.93	0.09
5	<0.05	3	689	0.96	0.10		0.95	0.10		18	0.99	0.09
6	<0.05	3	459	0.96	0.11		0.96	0.11		248	0.87	0.10
7	<0.05	3	698	0.95	0.10		0.95	0.10		9	0.98	0.05
8	<0.05	3	683	0.95	0.10		0.95	0.10		24	0.30	0.07
9	<0.05	3	674	0.95	0.10		0.95	0.10		33	0.95	0.11
10	<0.05	3	680	0.95	0.10		0.95	0.10		27	0.85	0.09
11	<0.05	3	657	0.95	0.10		0.95	0.10		50	0.82	0.08
12	<0.05	3	584	0.97	0.09		0.96	0.09		123	0.52	0.12
13	<0.05	3	695	0.95	0.10		0.95	0.10		12	0.98	0.05

**Table 4:** Results obtained from 10-fold cross validation of OPLS regression models predicting serum BHBA concentration from ^1^H NMR spectra using data from Farm 1 only, uncorrected data from all farms, and data from all farms corrected for the effect of herd. Number of cows (N), number of latent variables included in each mode (LV), coefficient of determination (R^2^) and root mean square error (RMSE) of calibration (C) and 10-fold cross validation (CV) are shown.

Dataset	N	LVs	P Value ^1^	R^2^_C_	RMSE_C_	R^2^_CV_	RMSE_CV_
Farm 1 Uncorrected	129	4	<0.001	0.98	0.10	0.97	0.12
All Data Uncorrected	707	4	<0.001	0.96	0.09	0.96	0.10
All Data Corrected for Herd	707	4	<0.001	0.93	0.09	0.93	0.09

^1^ P-value derived from permutation testing (50 iterations) and pairwise Wilcoxon signed rank test.

## Supplementary Materials

The following are available online at https://www.mdpi.com/2218-1989/10/5/180/s1, Table S1. ^1^H NMR chemical shifts (δ) and multiplicity of metabolites in bovine serum run in deuterated water (D2O).; Table S2. Results of ANOVA-simultaneous component analysis (ASCA) of uncorrected ^1^H NMR spectra of bovine serum.; Figure S1. Representative 700MHz ^1^H NMR spectrum (δ 0.4 to 9.0) of serum obtained from a Holstein–Friesian cow in early lactation.; Figure S2. Results of PCA of 707 ^1^H NMR spectra of serum obtained from dairy cows in early lactation, corrected for weeks in milk using linear regression; (a) PC 1 vs. PC 2 scores, (b) PC 1 vs. PC 3 scores, (c) PC 2 vs. PC 3 scores, (d) PC 1 loadings, (e) PC 2 loadings, and (f) PC 3 loadings plots.; Figure S3. Results of PCA of 707 ^1^H NMR spectra of serum obtained from dairy cows in early lactation, corrected for Parity using linear regression; (a) PC 1 vs. PC 2 scores, (b) PC 1 vs. PC 3 scores, (c) PC 2 vs. PC 3 scores, (d) PC 1 loadings, (e) PC 2 loadings, and (f) PC 3 loadings plots.; Figure S4. Results of PCA of 707 ^1^H NMR spectra of serum obtained from dairy cows in early lactation, corrected for Herd using linear regression; (a) PC 1 vs. PC 2 scores, (b) PC 1 vs. PC 3 scores, (c) PC 2 vs. PC 3 scores, (d) PC 1 loadings, (e) PC 2 loadings, and (f) PC 3 loadings plots.; Figure S5. Average ^1^H NMR spectrum of bovine serum. Color-coding represents the percentage of variation in the signal at each chemical shift intensity that can be explained by (a) WIM and (b) Parity: Figure S6: Results of OPLS regressions of serum BHBA concentration against ^1^H NMR spectrum of bovine serum (n = 707): (a) LV1 vs. LV2 scores for uncorrected data (b) CV predicted vs. measured BHBA (c) LV1 vs. LV2 scores for corrected data (d) CV predicted vs. measured corrected BHBA ranking.

## References

[1] Wishart D.S. (2008). Metabolomics: Applications to food science and nutrition research. Trends Food Sci. Technol..

[2] Drackley J.K. (1999). Biology of dairy cows during the transition period: The final frontier?. J. Dairy Sci..

[3] Grummer R.R. (1995). Impact of changes in organic nutrient metabolism on feeding the transition dairy cow. J. Anim. Sci..

[4] LeBlanc S.J., Lissemore K.D., Kelton D.F., Duffield T.F., Leslie K.E. (2006). Major advances in disease prevention in dairy cattle. J. Dairy Sci..

[5] Houle D., Govindaraju D., Omholt S. (2010). Phenomics: The next challenge. Nat. Rev. Gen..

[6] Daetwyler H.D., Xiang R., Yuan Z., Bolormaa S., Vander Jagt C.J., Hayes B.J., van der Werf J.H.J., Pryce J.E., Chamberlain A.J., Macleod I.M. Integration of functional genomics and phenomics into genomic prediction raises its accuracy in sheep and dairy cattle. Proceedings of the Association for the Advancement of Animal Breeding and Genetics.

[7] Xiang R., Berg I.V.D., Macleod I.M., Hayes B.J., Prowse-Wilkins C.P., Wang M., Bolormaa S., Liu Z., Rochfort S.J., Reich C.M. (2019). Quantifying the contribution of sequence variants with regulatory and evolutionary significance to 34 bovine complex traits. Proc. Natl. Acad. Sci. USA.

[8] Sun L.W., Zhang H.Y., Wu L., Shu S., Xia C., Xu C., Zheng J.S. (2014). 1H-Nuclear magnetic resonance-based plasma metabolic profiling of dairy cows with clinical and subclinical ketosis. J. Dairy Sci..

[9] Zhang G., Dervishi E., Dunn S., Mandal R., Liu P., Han B., Wishart D., Ametaj B. (2017). Metabotyping reveals distinct metabolic alterations in ketotic cows and identifies early predictive serum biomarkers for the risk of disease. Metabolomics.

[10] Sun Y., Xu C., Li C., Cheng X., Xu C., Wu L., Zhang H. (2014). Characterization of the serum metabolic profile of dairy cows with milk fever using (1)H-NMR spectroscopy. Vet. Quart..

[11] Basoglu A., Baspinar N., Tenori L., Licari C., Gulersoy E. (2020). Nuclear magnetic resonance (NMR)-based metabolome profile evaluation in dairy cows with and without displaced abomasum. Vet. Quart..

[12] Hailemariam D., Mandal R., Saleem F., Dunn S.M., Wishart D.S., Ametaj B.N. (2014). Identification of predictive biomarkers of disease state in transition dairy cows. J. Dairy Sci..

[13] Goldansaz S.A., Guo A.C., Sajed T., Steele M.A., Plastow G.S., Wishart D.S. (2017). Livestock metabolomics and the livestock metabolome: A systematic review. PLoS ONE.

[14] Sacco D., Brescia M.A., Sgaramella A., Casiello G., Buccolieri A., Ogrinc N., Sacco A. (2009). Discrimination between southern Italy and foreign milk samples using spectroscopic and analytical data. Food Chem..

[15] Tenori L., Santucci C., Meoni G., Morrocchi V., Matteucci G., Luchinat C. (2018). NMR metabolomic fingerprinting distinguishes milk from different farms. Food Res. Int..

[16] Sundekilde U.K., Frederiksen P.D., Clausen M.R., Larsen L.B., Bertram H.C. (2011). Relationship between the metabolite profile and technological properties of bovine milk from two dairy breeds elucidated by NMR-based metabolomics. J. Agric. Food Chem..

[17] Ilves A., Harzia H., Ling K., Ots M., Soomets U., Kilk K. (2012). Alterations in milk and blood metabolomes during the first months of lactation in dairy cows. J. Dairy Sci..

[18] Maher A.D., Hayes B., Cocks B., Marett L., Wales W.J., Rochfort S.J. (2013). Latent biochemical relationships in the blood-milk metabolic axis of dairy cows revealed by statistical integration of 1H NMR spectroscopic data. J. Proteome Res..

[19] Mrode R.A. (2014). Linear Models for the Prediction of Animal Breeding Values.

[20] Wanichthanarak K., Jeamsripong S., Pornputtapong N., Khoomrung S. (2019). Accounting for biological variation with linear mixed-effects modelling improves the quality of clinical metabolomics data. Comput. Struct. Biotechnol. J..

[21] Laine A., Bastin C., Grelet C., Hammami H., Colinet F.G., Dale L.M., Gillon A., Vandenplas J., Dehareng F., Gengler N. (2017). Assessing the effect of pregnancy stage on milk composition of dairy cows using mid-infrared spectra. J. Dairy Sci..

[22] Oetzel G.R. (2004). Monitoring and testing dairy herds for metabolic disease. Vet. Clin. North Am. Food Anim. Pr..

[23] Ospina P.A., Nydam D.V., Stokol T., Overton T.R. (2010). Associations of elevated nonesterified fatty acids and β-hydroxybutyrate concentrations with early lactation reproductive performance and milk production in transition dairy cattle in the northeastern United States. J. Dairy Sci..

[24] Lean I., Westwood C.T., Playford M.C. (2008). Livestock disease threats associated with intensification of pastoral dairy farming. N. Z. Vet. J..

[25] Schwaiger T., Beauchemin K.A., Penner G.B. (2013). Duration of time that beef cattle are fed a high-grain diet affects the recovery from a bout of ruminal acidosis: Short-chain fatty acid and lactate absorption, saliva production, and blood metabolites. J. Anim. Sci..

[26] Yang Y., Dong G., Wang Z., Wang J., Zhang Z., Liu J. (2018). Rumen and plasma metabolomics profiling by UHPLC-QTOF/MS revealed metabolic alterations associated with a high-corn diet in beef steers. PLoS ONE.

[27] Zhang L., Martins A.F., Zhao P., Tieu M., Esteban-Gómez D., McCandless G.T., Platas-Iglesias C., Sherry A.D. (2017). Enantiomeric recognition of d- and l-lactate by CEST with the aid of a paramagnetic shift reagent. J. Am. Chem. Soc..

[28] Lees H.J., Swann J.R., Wilson I.D., Nicholson J.K., Holmes E. (2013). Hippurate: The natural history of a mammalian–microbial cometabolite. J. Proteome Res..

[29] Carpio A., Bonilla-Valverde D., Arce C., Rodríguez-Estévez V., Sánchez-Rodríguez M., Arce L., Valcárcel M. (2013). Evaluation of hippuric acid content in goat milk as a marker of feeding regimen. J. Dairy Sci..

[30] Pallister T., Jackson M.A., Martin T.C., Zierer J., Jennings A., Mohney R.P., MacGregor A., Steves C.J., Cassidy A., Spector T.D. (2017). Hippurate as a metabolomic marker of gut microbiome diversity: Modulation by diet and relationship to metabolic syndrome. Sci. Rep..

[31] Liao Y., Hu R., Wang Z., Peng Q., Dong X., Zhang X., Zou H., Pu Q., Xue B., Wang L. (2018). Metabolomics profiling of serum and urine in three beef cattle breeds revealed different levels of tolerance to heat stress. J. Agric. Food Chem..

[32] Yin P., Lehmann R., Xu G. (2015). Effects of pre-analytical processes on blood samples used in metabolomics studies. Anal. Bioanal. Chem..

[33] Beckonert O., Keun H.C., Ebbels T.M., Bundy J., Holmes E., Lindon J.C., Nicholson J.K. (2007). Metabolic profiling, metabolomic and metabonomic procedures for NMR spectroscopy of urine, plasma, serum and tissue extracts. Nat. Protoc..

[34] Emwas A.-H., Luchinat C., Turano P., Tenori L., Roy R., Salek R.M., Ryan D., Merzaban J.S., Kaddurah-Daouk R., Zeri A.C. (2015). Standardizing the experimental conditions for using urine in NMR-based metabolomic studies with a particular focus on diagnostic studies: A review. Metabolomics.

[35] Jobard E., Trédan O., Postoly D., André F., Martin A.-L., Elena-Herrmann B., Boyault S. (2016). A systematic evaluation of blood serum and plasma pre-analytics for metabolomics cohort studies. Int. J. Mol. Sci..

[36] Klein M.S., Almstetter M.F., Schlamberger G., Nürnberger N., Dettmer K., Oefner P.J., Meyer H.H.D., Wiedemann S., Gronwald W. (2010). Nuclear magnetic resonance and mass spectrometry-based milk metabolomics in dairy cows during early and late lactation. J. Dairy Sci..

[37] Cozzi G., Ravarotto L., Gottardo F., Stefani A.L., Contiero B., Moro L., Brscic M., Dalvit P. (2011). Short communication: Reference values for blood parameters in holstein dairy cows: Effects of parity, stage of lactation, and season of production. J. Dairy Sci..

[38] Luke T.D.W., Rochfort S., Wales W.J., Bonfatti V., Marett L., Pryce J.E. (2019). Metabolic profiling of early-lactation dairy cows using milk mid-infrared spectra. J. Dairy Sci..

[39] Morales Pineyrua J.T., Farina S.R., Mendoza A. (2018). Effects of parity on productive, reproductive, metabolic and hormonal responses of Holstein cows. Anim. Reprod. Sci..

[40] Davies A., Fearn T. (2006). Back to basics: Calibration statistics. Spectrosc. Eur..

[41] Posma J.M., Garcia-Perez I., Ebbels T.M.D., Lindon J.C., Stamler J., Elliott P., Holmes E., Nicholson J.K. (2018). Optimized phenotypic biomarker discovery and confounder elimination via covariate-adjusted projection to latent structures from metabolic spectroscopy data. J. Proteome Res..

[42] Lean I.J., Farver T.B., Troutt H.F., Bruss M.L., Galland J.C., Baldwin R.L., Holmberg C.A., Weaver L.D. (1992). Time series cross-correlation analysis of postparturient relationships among serum metabolites and yield variables in Holstein cows. J. Dairy Sci..

[43] Aschenbach J.R., Kristensen N.B., Donkin S.S., Hammon H.M., Penner G.B. (2010). Gluconeogenesis in dairy cows: The secret of making sweet milk from sour dough. IUBMB Life.

[44] Drackley J.K., Overton T.R., Douglas G.N. (2001). Adaptations of glucose and long-chain fatty acid metabolism in liver of dairy cows during the periparturient period. J. Dairy Sci..

[45] Van den Berg R.A., Hoefsloot H.C.J., Westerhuis J.A., Smilde A.K., van der Werf M.J. (2006). Centering, scaling, and transformations: Improving the biological information content of metabolomics data. BMC Genom..

[46] Emwas A.-H., Saccenti E., Gao X., McKay R., Santos V., Roy R., Wishart D. (2018). Recommended strategies for spectral processing and post-processing of 1D 1 H-NMR data of biofluids with a particular focus on urine. Off. J. Metab. Soc..

[47] Fontanesi L. (2016). Metabolomics and livestock genomics: Insights into a phenotyping frontier and its applications in animal breeding. Anim. Front..

[48] Boichard D., Brochard M. (2012). New phenotypes for new breeding goals in dairy cattle. Anim. Int. J. Anim. Biosci..

[49] Egger-Danner C., Cole J.B., Pryce J.E., Gengler N., Heringstad B., Bradley A., Stock K.F. (2015). Invited review: Overview of new traits and phenotyping strategies in dairy cattle with a focus on functional traits. Anim. Int. J. Anim. Biosci..

[50] De Haas Y., Pryce J.E., Calus M.P., Wall E., Berry D.P., Lovendahl P., Krattenmacher N., Miglior F., Weigel K., Spurlock D. (2015). Genomic prediction of dry matter intake in dairy cattle from an international data set consisting of research herds in Europe, North America, and Australasia. J. Dairy Sci..

[51] McMurray C.H., Blanchflower W.J., Rice D.A. (1984). Automated kinetic method for D-3-hydroxybutyrate in plasma or serum. Clin. Chem..

[52] Gowda N.G.A., Raftery D. (2014). Quantitating metabolites in protein precipitated serum using NMR spectroscopy. Anal. Chem..

[53] Viant M.R. (2003). Improved methods for the acquisition and interpretation of NMR metabolomic data. Biochem. Biophys. Res. Commun..

[54] Nielsen N.-P.V., Carstensen J.M., Smedsgaard J. (1998). Aligning of single and multiple wavelength chromatographic profiles for chemometric data analysis using correlation optimised warping. J. Chromatogr..

[55] Eigenvector (2017). PLS Toolbox, R2017b.

[56] Team R.C. (2019). R: A Language and Environment for Statistical Computing.

[57] Wei T., Simko V. R Package “Corrplot”: Visualization of a Correlation Matrix, (Version 0.84). https://github.com/taiyun/corrplot.

[58] Gilmour A.R., Gogel B.J., Cullis B.R., Thompson R. (2015). ASReml User Guide Release 4.1 Functional Specification, 4.1.

[59] Smilde A., Jansen J., Hoefsloot H., Lamers R.-J., van Der Greef J., Timmerman M. (2005). ANOVA-simultaneous component analysis (ASCA): A new tool for analyzing designed metabolomics data. Bioinformatics.

[60] Chong I.-G., Jun C.-H. (2005). Performance of some variable selection methods when multicollinearity is present. Chemom. Intellig. Lab. Syst..

