# Supplementary material for: Use of Large and Diverse Datasets for 1H NMR Serum Metabolic Profiling of Early Lactation Dairy Cows
Source: Metabolites. 2020 Apr 30;10(5):180. doi: 10.3390/metabo10050180 (PMC7281003; doi:10.3390/metabo10050180)
Supplement: Supplementary file 1 [file metabolites-10-00180-s001.pdf]

metabolites

MDPI

Supplementary Material

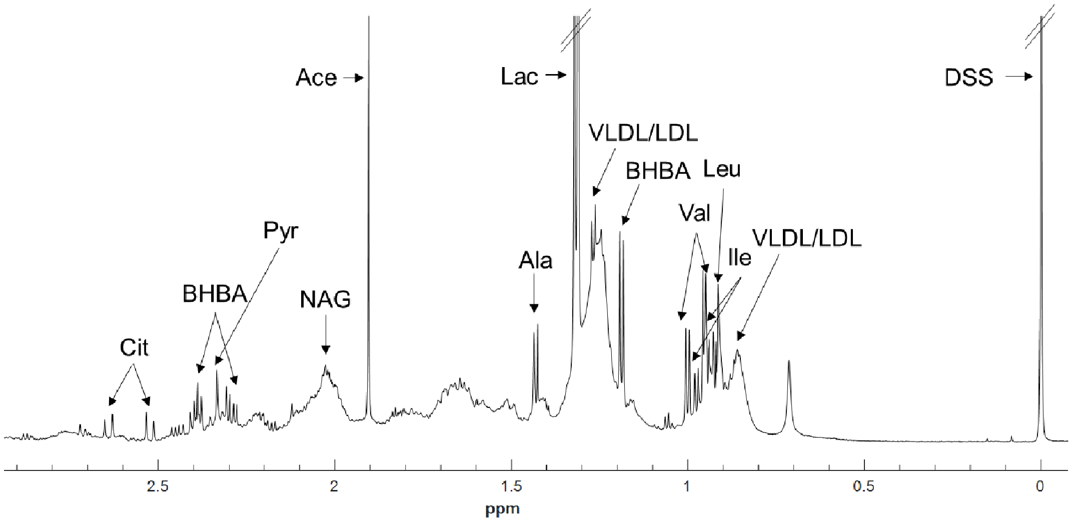

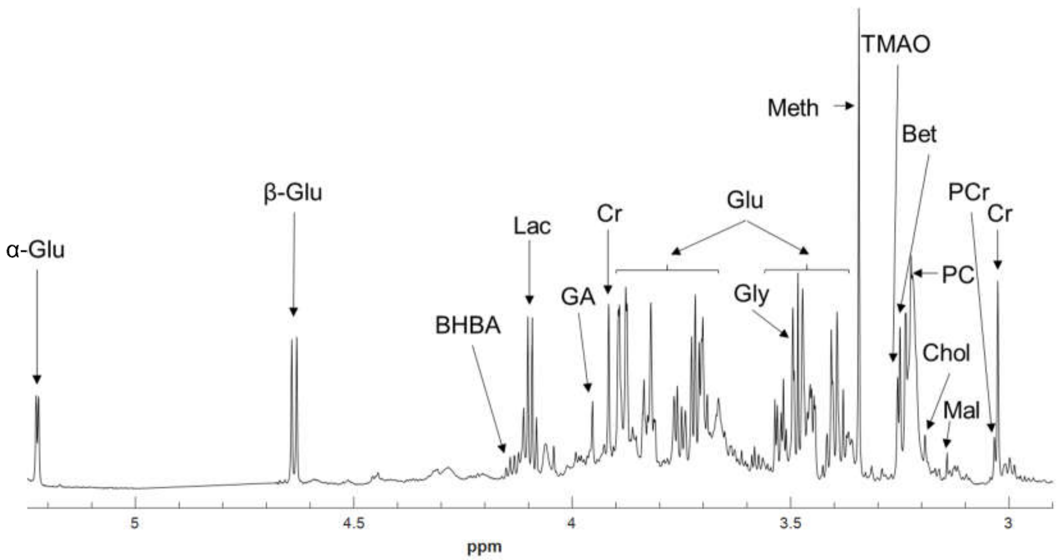

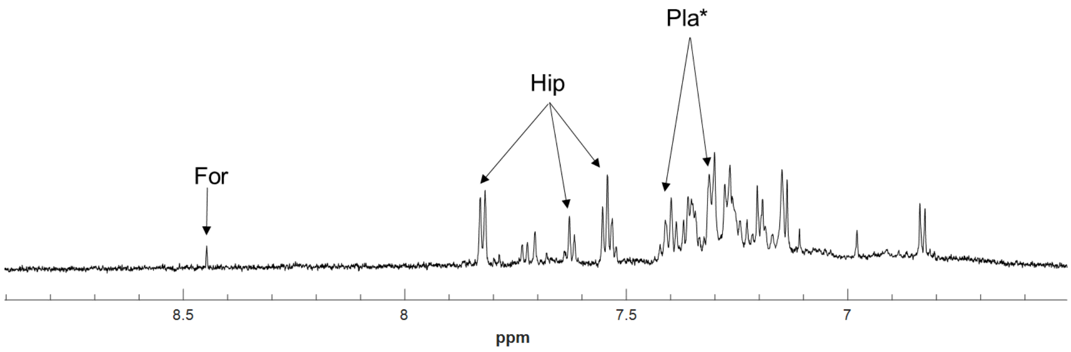

Figure S1. Representative 700MHz ^{1}H NMR spectrum (δ -0.1 to 9.0) of serum obtained from a Holstein-Friesian cow in early lactation. The δ 0.4 to 2.9, δ 2.9 to 5.25 and δ 6.5 to 9.0 regions have been magnified for clarity purposes. α-Glu = α glucose, β-Glu = β glucose, Ace = acetate, Ala = alanine, Bet = betaine, BHBA = β hydroxybutyrate, Cit = citrate, Chol = choline, Cr = creatine, For = formate, GA = glycolate, Glu = glucose, Gly = glycine, Hip = hippurate, Ile = isoleucine, Lac = lactate, Leu = leucine, Mal = malonate, Meth = methanol, NAG = N-acetyl glycoprotein, PC = phosphocholine, PCr = phosphocreatine, PLa* = 3-phenyllactate, Pyr = pyruvate, TMAO = Trimethylamine N-oxide, Val = valine, VLDL/LDL = Very low density lipoprotein and low density lipoprotein. * = tentative identification.

Table S1. ^{1}H NMR chemical shifts (δ) and multiplicity of metabolites in bovine serum run in deuterated water (D_2O). Clearly observed resonances are indicated in bold text**. s**, singlet; d, doublet; dd, doublet of a doublet; m, multiplet; t, triplet.

Label	Metabolite	Chemical shift (δ) and multiplicity
Leu	leucine	**0.94 (d)**, **0.95 (d)**, 1.66 (m), 1.66 (m), 1.73 (m), 3.72 (m)
Ile	isoleucine	**0.93 (t)**, **1.00 (d)**, 1.24 (m), 1.45 (m), 1.45 (m), 3.66 (d)
Val	valine	**0.98 (d)**, **1.03 (d)**, 2.26 (m), 3.60 (d)
BHBA	β-hydroxybutyrate	**1.20 (d)**, **2.31 (m), 2.41 (m), 4.16 (m)**
Lac	lactate	**1.31 (d),** 4.31 (q)
Ala	alanine	**1.46 (d),** 3.77 (q)
Ace	acetate	**1.9 (s)**
Pyr	pyruvate	**2.46 (s),** 7.65 (s)
Cit	citrate	**2.52 (d), 2.66 (d)**
Cr	creatine	**3.02 (s), 3.92 (s)**
PCr	phosphocreatine	**3.03 (s)**, **3.93 (s)**
Mal	malonate	**3.11 (s)**
Chol	choline	**3.19 (s)**, 3.50 (m), 4.05 (m)
PC	phosphocholine	**3.21 (s)**, 3.58 (t), 4.17 (m)
Bet	betaine	**3.25 (s)**, 3.89 (s)
TMAO	trimethylamine N-oxide	**3.25 (s)**
Meth	methanol	**3.34 (s)**
Glu	glucose	**3.23 (dd)**, **3.40 (m), 3.46 (m), 3.52 (dd), 3.73 (m), 3.82 (m), 3.89 (dd), 4.63 (d)**, **5.22 (d)**
Gly	glycine	**3.50 (s)**
GA	glycolate	**3.93 (s)**
β-Glu	β-glucose	**4.63 (d)**
α-Glu	α-glucose	**5.22 (d)**
PLa	3-phenyllactate*	**2.87 (dd)**, 3.09 (dd), 4.26 (dd), **7.31 (m), 7.39 (m)**
Hip	hippurate	3.96 (d), **7.54 (m), 7.62 (m), 7.83 (dd)**

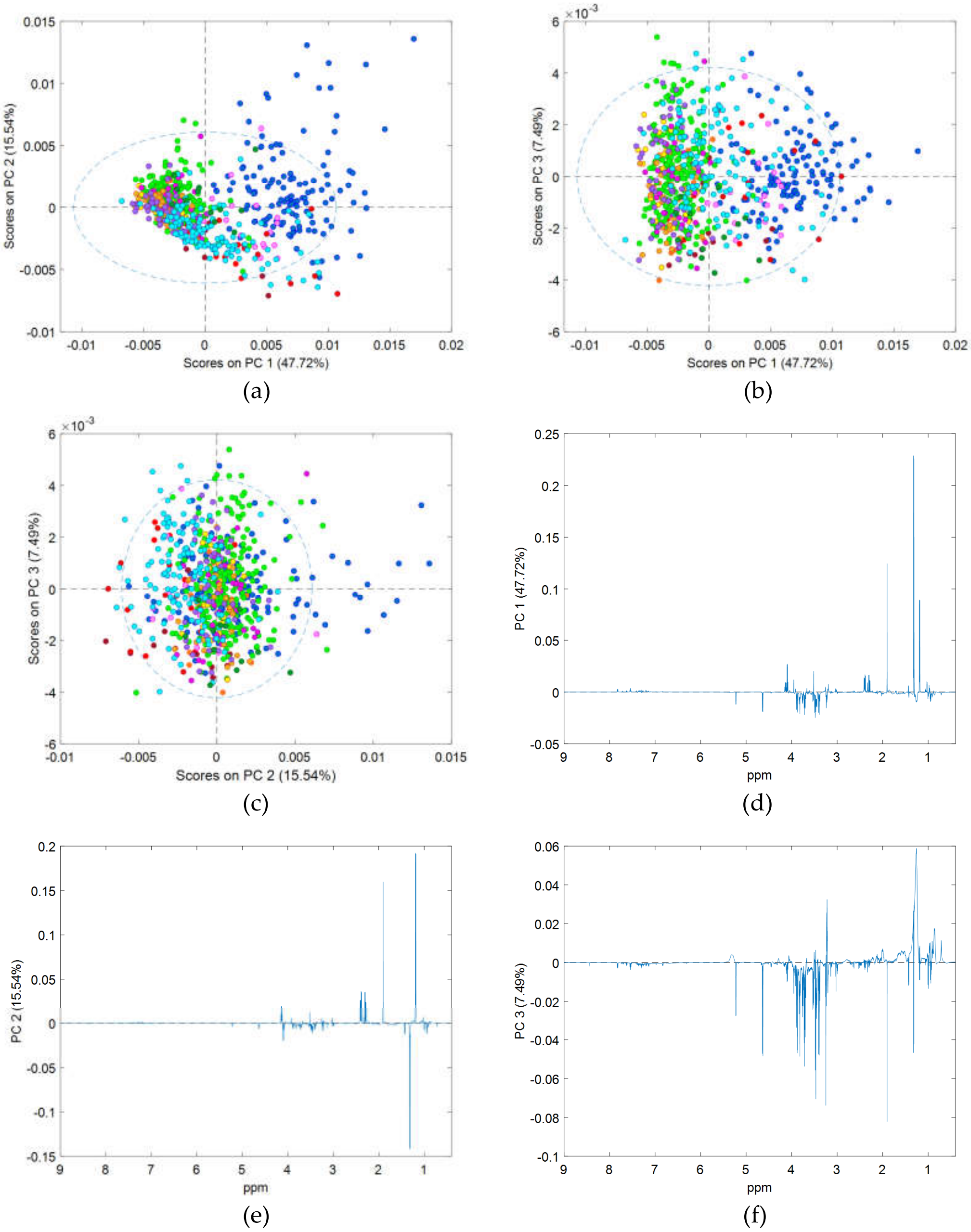

Figure S2. Results of PCA of 707 ^{1}H NMR spectra of serum obtained from dairy cows in early lactation, corrected for weeks in milk using linear regression; (a) PC 1 vs PC 2 scores, (b) PC 1 vs PC 3 scores (c) PC 2 vs PC 3 scores (d) PC 1 loadings (e) PC 2 loadings and (f) PC 3 loadings plots. Scores plots are coloured by farm of origin.

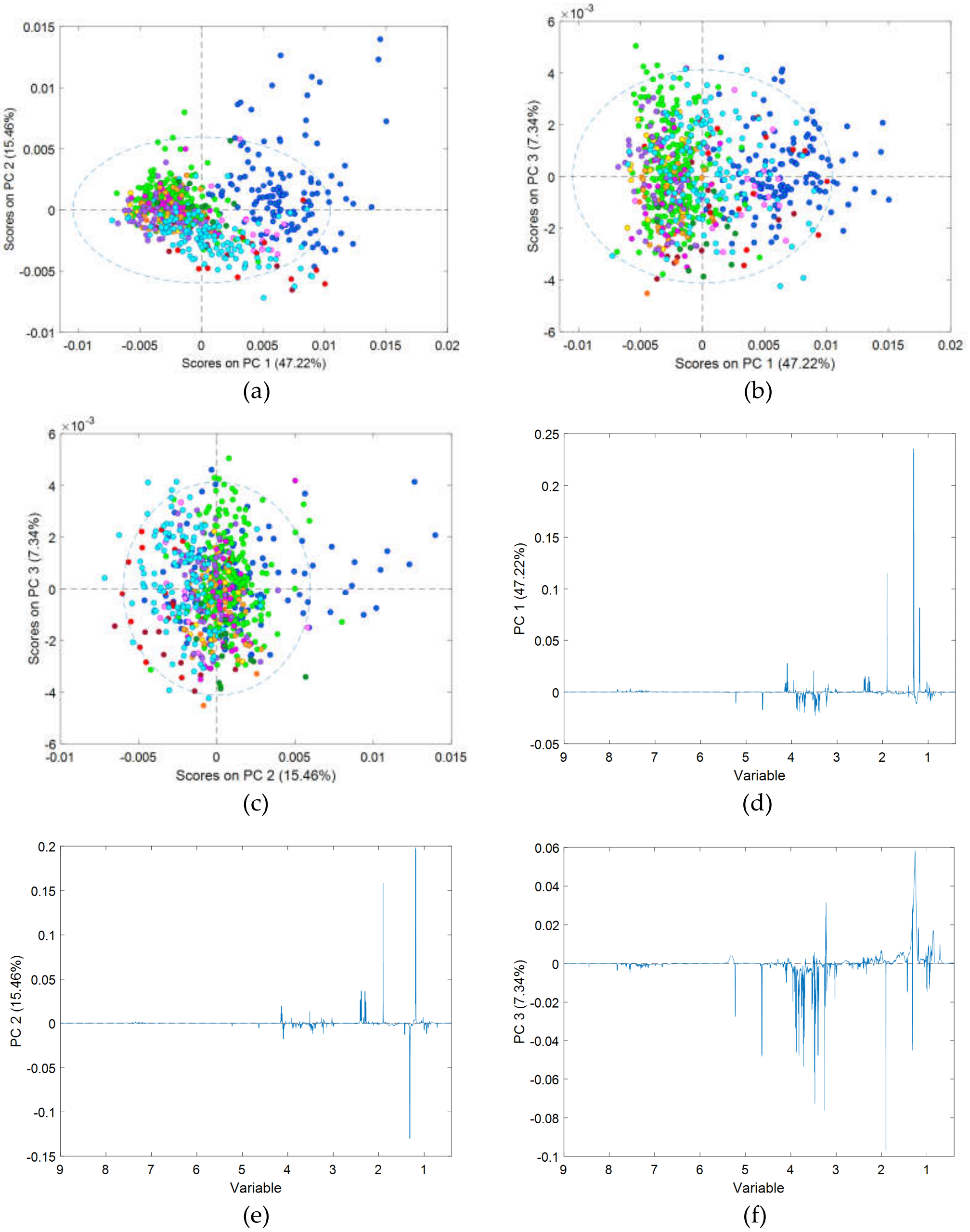

Figure S3. Results of PCA of 707 ^{1}H NMR spectra of serum obtained from dairy cows in early lactation, corrected for Parity using linear regression; (a) PC 1 vs PC 2 scores, (b) PC 1 vs PC 3 scores (c) PC 2 vs PC 3 scores (d) PC 1 loadings (e) PC 2 loadings and (f) PC 3 loadings plots. Scores plots are coloured by farm of origin.

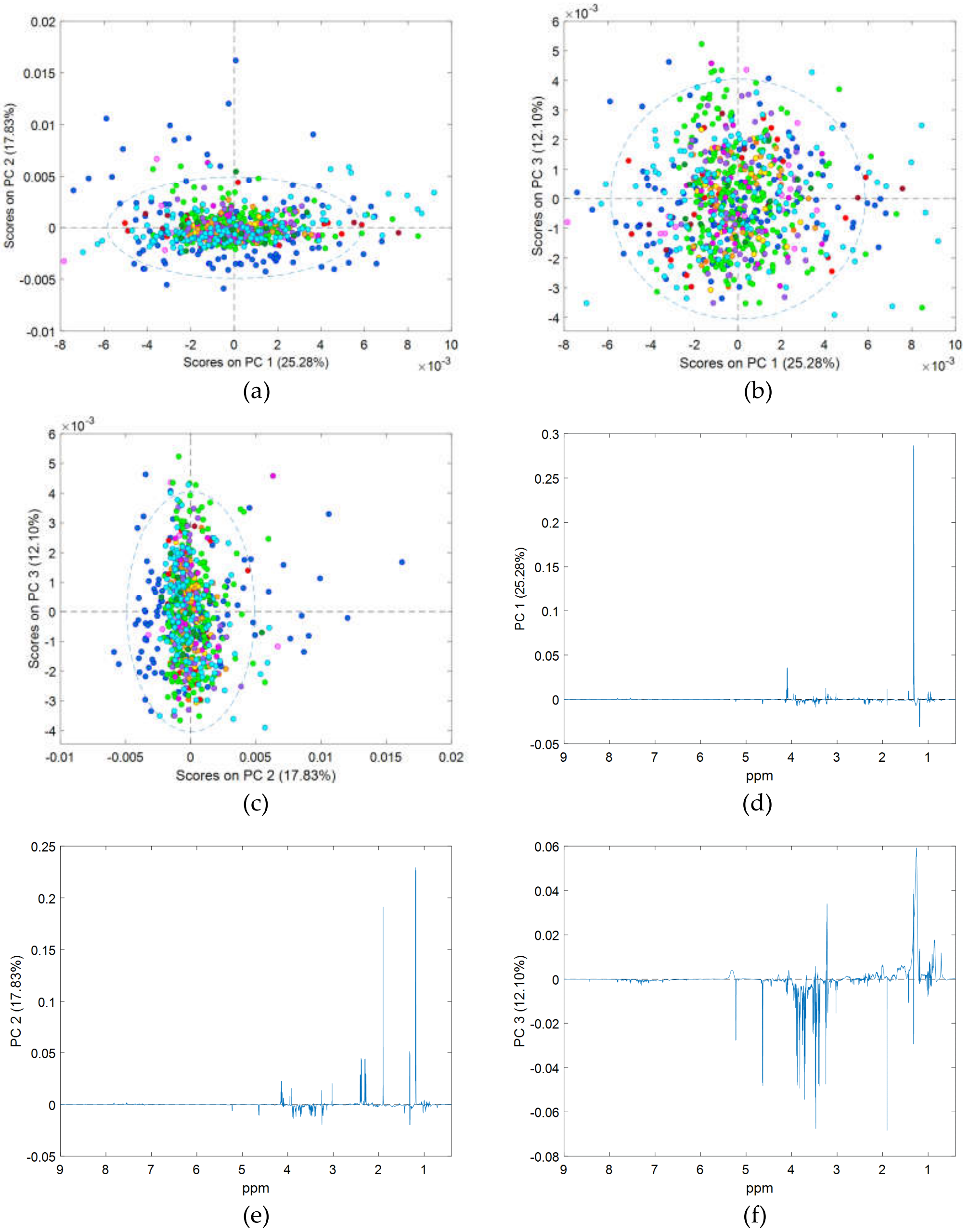

Figure S4. Results of PCA of 707 ¹H NMR spectra of serum obtained from dairy cows in early lactation, corrected for Herd using linear regression; (a) PC 1 vs PC 2 scores, (b) PC 1 vs PC 3 scores (c) PC 2 vs PC 3 scores (d) PC 1 loadings (e) PC 2 loadings and (f) PC 3 loadings plots. Scores plots are coloured by farm of origin.

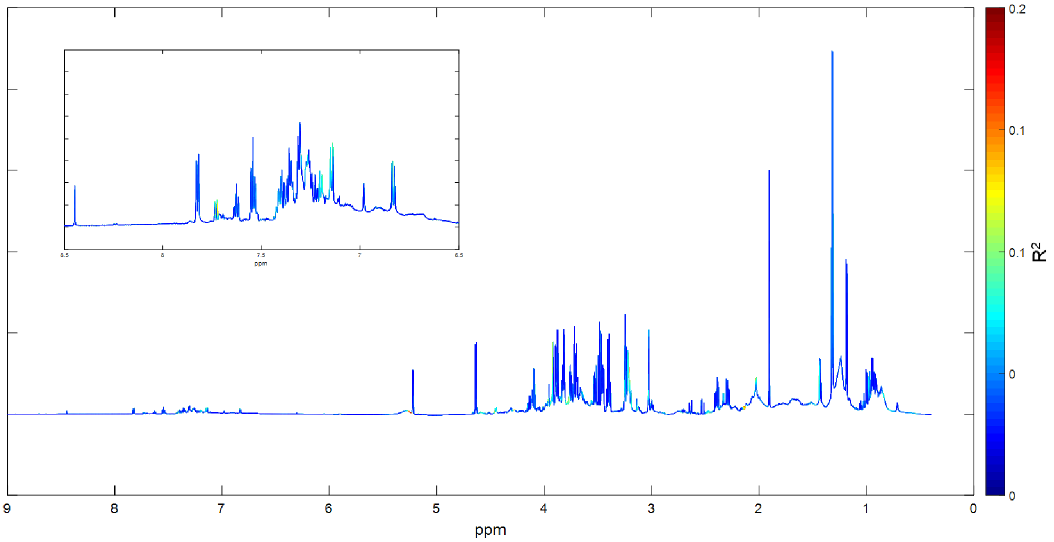

(a)

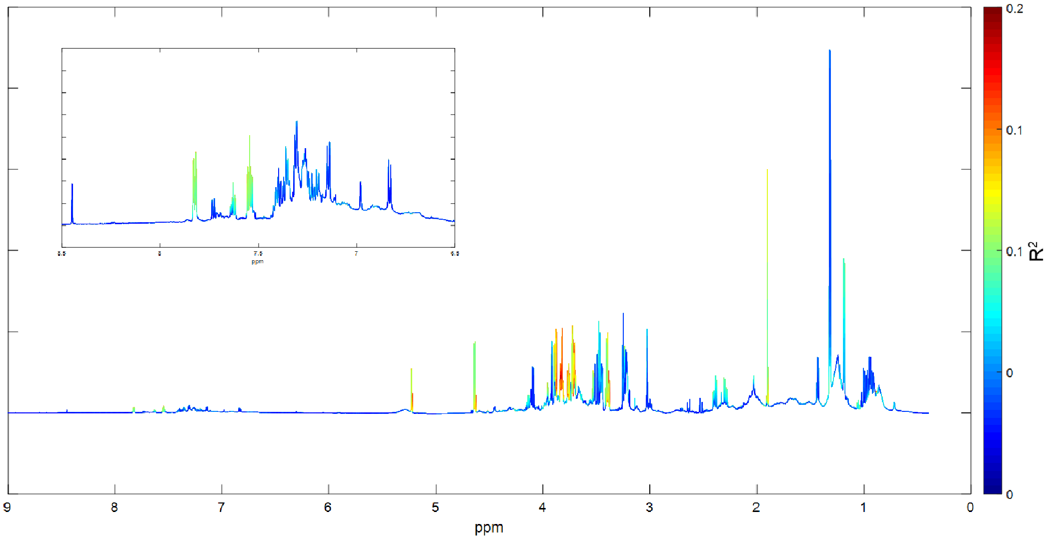

(b)

Figure S5. Average ^{1}H NMR spectrum of bovine serum. Colour-coding represents the percentage of variation in the signal at each chemical shift intenisty that can be explained by (a) WIM and (b) Parity. The δ 6.5 to 8.5 region has been magnified for clarity purposes.

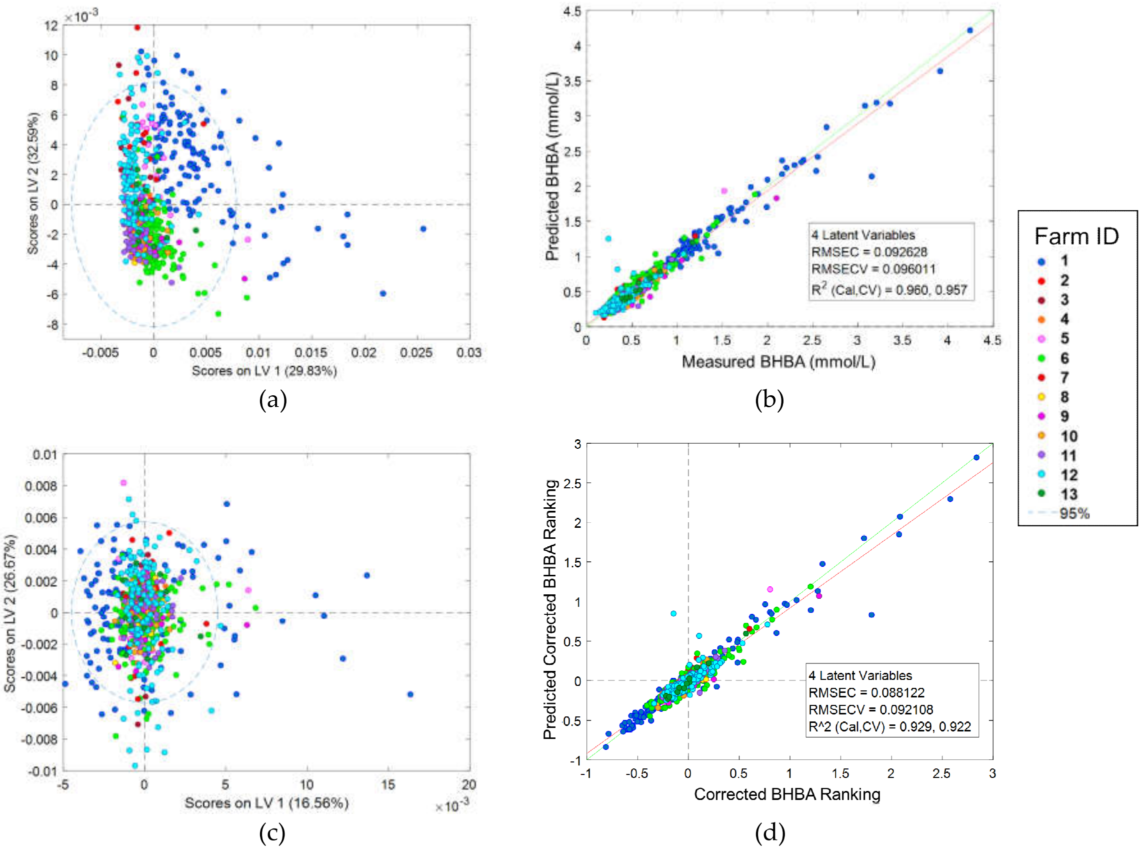

Figure S6. Results of OPLS regressions of serum BHBA concentration against ^{1}H NMR spectrum of bovine serum (n = 707): (a) LV1 vs LV2 scores for uncorrected data (b) CV predicted vs measured BHBA (c) LV1 vs LV2 scores for data corrected for all fixed effects (d) CV predicted vs measured corrected BHBA ranking. Plots are colored by Herd.

Table S2. Results of ANOVA-simultaneous coponent analysis (ASCA) of uncorrected ^{1}H NMR spectra of bovine serum (n = 707), and spectra that have been corrected using linear regression for (1) all fixed effects, (2) weeks in milk (WIM), (3) parity, and (4) herd of origin. Effect describes the relative influence of each variable (weeks in milk (WIM), Parity and Herd) on each spectral dataset. P-value is derived from permutation testing (50 iterations).

	Uncorrected		All Fixed Effects		WIM		Parity		Herd	
Variable	Effect	P-Value	Effect	P-Value	Effect	P-Value	Effect	P-Value	Effect	P-Value
WIM	1.37	0.02	0.00	1.00	0.00	0.02	1.55	0.02	1.68	0.02
Parity	4.10	0.02	0.00	1.00	4.24	0.02	0.00	0.02	3.30	0.02
Herd	43.99	0.02	0.00	1.00	43.60	0.02	41.72	0.02	0.00	1.00
